# Ir-192 Calibration in Air with Farmer Chamber for HDR Brachytherapy

**DOI:** 10.1007/s40846-016-0117-0

**Published:** 2016-03-31

**Authors:** Liyun Chang, Sheng-Yow Ho, Tsair-Fwu Lee, Hueisch-Jy Ding, Pang-Yu Chen

**Affiliations:** Department of Medical Imaging and Radiological Sciences, I-Shou University, Kaohsiung, 82445 Taiwan; Department of Nursing, Chang Jung Christian University, Tainan, 71101 Taiwan; Department of Radiation Oncology, Chi Mei Medical Center, Liouying, Tainan, 73657 Taiwan; Medical Physics and Informatics Laboratory, Department of Electronics Engineering, National Kaohsiung University of Applied Sciences, Kaohsiung, 80778 Taiwan; Department of Radiation Oncology, Sinlau Christian Hospital, Tainan, 70142 Taiwan

**Keywords:** Ir-192 calibration, In-air calibration, Farmer-type ion chamber, High dose rate (HDR)

## Abstract

A comprehensive review for the in-air calibration of an Ir-192 high-dose-rate brachytherapy source in terms of air kerma strength (AKS) and reference air kerma rate (RAKR) using the Farmer chamber was conducted. The reviewed calibration methods include the National Physical Laboratory (NPL) calibration standard in the UK, the 7-distance technique with the standard calibration of the National Institute of Standards and Technology and Accredited Dosimetry Calibration Laboratory in the US, the calibration conducted in Australia following recommendations of the International Atomic Energy Agency with the chamber primarily calibrated by the Australian Radiation Protection and Nuclear Safety Agency, the calibration conducted in India following the Deutsche Gesellschaft fur Medizinische Physik recommendation, and the convenient empirical method used in Taiwan. The calibrated AKS (or RAKR) and uncertainty obtained using Farmer chambers are similar to those obtained using well chambers. All reported differences (between measurements obtained using Farmer and well chambers, respectively) and uncertainties (*k* = 2) were generally less than 1.5 and 2.5 %, respectively. The standard uncertainty of the NPL calibration is approximately half that of all the other proposed approaches, and may become the gold standard calibration procedure. Almost all techniques follow the 7-distance technique basis; however, the services at NPL can calibrate the source with lower uncertainty. Users can calibrate the Ir-192 source more conveniently using the empirical method with only one source-chamber distance.

## Introduction

A clinical high-dose-rate (HDR) brachytherapy system is usually equipped with an Ir-192 source, which has a decay half-life of 73.83–74.02 days [1]. The activity of a new Ir-192 source is generally around 10 Ci, which in the units of air kerma strength (AKS) or reference air kerma rate (RAKR) is around 1.1337 × 10^−5^ Gy·m^2^·s^−1^ [2, 3]. To avoid prolonging treatment, it is necessary to replace the source approximately four times a year. Each time a new HDR source is installed for use in clinical routine, it is essential that a source calibration in the facility be carried out. The calibration procedure is the main component of quality assurance (QA) programs for HDR brachytherapy [4–6].

**Fig. 1:**
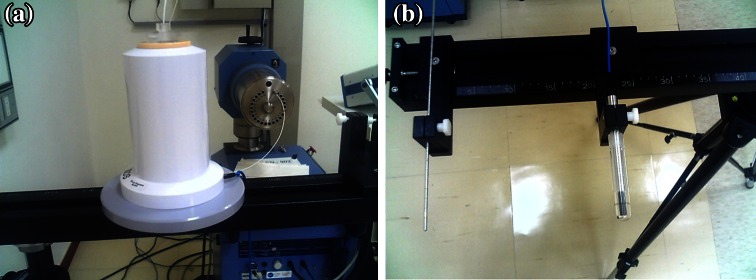
Calibration of HDR Ir-192 using **a** well chamber and **b** Farmer chamber with calibration jig

Various techniques for calibrating Ir-192 have been developed [7–12]. These include the use of well-type ion chambers (Fig. 1a), Farmer-type ion chambers with a calibration jig (Fig. 1b), or other commercial devices [13]. Generally speaking, it is more complicated and cumbersome to do the source calibration using a Farmer-type chamber than a well-type chamber; therefore, a well chamber is usually preferred by medical physicists for performing calibration [14]. However, in some hospitals, a well-type ion chamber may not be available due to budgetary or other considerations. Furthermore, a Farmer chamber calibrated at the National Institute of Standards and Technology (NIST), Primary Standard Dosimetry Laboratory (PSDL), or Accredited Dosimetry Calibration Laboratory (ADCL) has a better calibration factor accuracy (1–2 %) than that of a well chamber (2–3 %) [14]. The one exception is if the calibration is conducted by the National Physical Laboratory (NPL) in the UK; the expended uncertainty of the calibration coefficient obtained using the well chamber (0.8 %) is slightly lower than that obtained using the Farmer chamber/jig combination (1.1 %) [3, 15]. In principle, more accurate calibration results can be obtained by using the Farmer chamber compared to those obtained using the well chamber [14, 16, 17].

The calibration of an Ir-192 HDR system using the Farmer chamber has been studied for decades [8–12]. The standard AKS calibration for Ir-192 sources was established at the University of Wisconsin Accredited Dosimetry Calibration Laboratory (UWADCL) in 1991 by Goetsch et al. [9], who employed an interpolation procedure using NIST calibrations of 662 keV (Cs-137) and 146 keV (250 kVcp X-rays) to get a calibration of 397 keV (the exposure-weighted average Ir-192 energy) because NIST does not offer calibration of ionization chambers with the gamma-ray spectrum of Ir-192. This calibration technique, also known as the 7-distance measurement technique, is still employed in most clinics, in which the source output at 7 source-to-chamber distances is measured and the room scatter factor, an important component of the calibration, is determined using a fitting process [9, 12]. One can obtain the RAKR calibration coefficient of Ir-192 using the services of NPL in the UK [18], removing the need for an interpolation procedure. The units of AKS and RAKR have the same physical quantity; the difference between them is that the dose and time units of RAKR are in Gy and seconds [3]. The 7-distance technique has been combined with other calibration techniques, such as the calibration conducted in Australia by Butler et al. [18] following recommendations of the International Atomic Energy Agency (IAEA) with the chamber primarily calibrated by the Australian Radiation Protection and Nuclear Safety Agency (ARPANSA), and the calibration conducted in India by Patel et al. [16] and Bondel et al. [17] following the Deutsche Gesellschaft fur Medizinische Physik (DGMP) recommendation.

However, it is cumbersome to perform a calibration using a Farmer chamber with a calibration jig, which is necessary for the 7-distance technique. The data are acquired with the source-chamber distance set at various values (distances are accurately measured), with curve fitting performed to obtain the room scatter. To overcome the difficulties of obtaining the room scatter, Selvam et al. derived the room scatter for brachytherapy treatment rooms of various sizes using Monte Carlo methods [19]. Using the published data of Selvam et al. in combination with experimental results, Chang et al. developed an empirical formula for directly calculating the room scatter for a concrete treatment room of almost any size [14].

Regardless of which type of detector is used, the standard uncertainty of source calibration in terms of AKS should be within ± 5 % (*k* = 1) [20–24], or even more stringently within ± 3 % (*k* = 1) [23]. Due to budgetary limitations, a calibration jig may not be available in some treatment facilities. In this case, the source may be calibrated with a Farmer-type ion chamber, but the associated scattering may be ignored. If room scatter is neglected (which increases calibrated uncertainty when the source-to-chamber distance is increased [16, 24]) with the assumed 2-mm measurement error of the source-to-chamber distance, according to the study of Chang et al. [14], for a room size of 4 × 4 × 4 m^3^, the minimum theoretical combined error is around 2.8 %. Without the use of a calibration jig, Chang et al. devised a practical technique for calibrating an Ir-192 source for HDR brachytherapy that can be easily implemented and reduces the measurement error of the source-to-chamber distance [25]. Compared to the traditional 7-distance technique [9, 12], this technique uses tools commonly employed for the QA of linear accelerators and needs only one measurement, obtained using an empirical formula with correction for the room scatter effect.

## Calibration Based on NPL Air Kerma Standard in United Kingdom

To obtain the RAKR (in terms of Gy·s^−1^ at 1 m), the NPL has established a primary standard cavity chamber, a spherical graphite-walled ionization chamber, as a primary standard for Ir-192. The Nucletron microSelectron Classic Ir-192 HDR source is used. The NPL provides HDR brachytherapy calibration services that include the calibration of Farmer chambers, but the customer must provide the calibration jig as well as the Farmer chamber. The calibration procedure for a Farmer chamber with a jig is similar to that for the well chamber in the NPL, which is described below.

In the NPL, the customer’s Farmer chamber is first connected to the microSelectron. After the calibration setup with the jig is completed, the point of maximum chamber response (i.e., the sweet spot) of the customer’s Farmer chamber is found by stepping the Ir-192 source through the catheter, which is parallel to the long axis of the chamber, with the corrected ionization current versus dwell position of the source plotted. The RAKR calibration coefficient N_K_NPL_ (Gy/C) is the ratio of the known primary standard measurement of RAKR to the ionization current of the user’s chamber measured with the source placed at the sweet spot with the ion recombination correction, and is given by:

1 $$ N_{k\_NPL} = \frac{{P_{NPL} (Gy\;S^{ - 1} )}}{{I_{U} (A) \cdot k_{ion} }} $$

where *P*_*NPL*_ is the known primary standard measurement of RAKR, *I*_*U*_ is the measured ionization current of the customer’s chamber, and *k*_*ion*_ is the ion recombination correction, which is the reciprocal of *A*_*ion*_ (ion collection efficiency). *k*_*ion*_ can be determined using the two-voltage technique [26]:

2 $$ k_{ion} = \left( {\frac{4}{3} - \frac{{I_{{300{\text{V}}}} }}{{3 \cdot I_{{150{\text{V}}}} }}} \right)^{ - 1} $$

where *I*_300V_ and *I*_150V_ are the electrometer (or dosimeter) response in amps with the bias set to 300 and 150 V, respectively.

The primary standard calibration for Ir-192 in the NPL is based on Bragg-Gray theory, large cavity theory, and the measured ionization current from the spherical graphite-walled ionization chamber. With the same source-chamber setup as that used in the NPL, users can calculate their own RAKR using the following equation:

3 $$ \dot{K}_{U} = I_{U} \cdot k_{ion} \cdot N_{k\_NPL} $$

where $\dot{K}_{U}$ s the RAKR of the hospital source (Gy·s^−1^), *I*_*U*_ is the corrected ionization current (A) measured by the user, *k*_*ion*_ is the ion recombination correction factor, and *N*_*K_NPL*_ is the calibration coefficient of the ionization chamber (Gy·C^−1^) acquired from the NPL using Eq. (1).

According to the report of Bidmead et al., the total uncertainties of the Farmer (thimble) chamber and well chamber calibration coefficients determined at the NPL are 1.1 % (*k* = 2, i.e., 2σ) and 0.8 % (*k* = 2), respectively, with the greater uncertainty of the Farmer chamber mainly due to the setup positional uncertainty of the Farmer chamber in the calibration jig [3].

## Calibration Based on Traditional 7-Distance Method

The photon spectrum of Ir-192 in an HDR unit includes approximately 24 1ines in the energy range of 9–885 keV. With the consideration that approximately 88 % of the exposure is delivered by 12 gamma lines at or above 296 keV and that the two strong L X-ray lines at 9.00 and 9.44 keV, respectively, are almost completely attenuated by the source capsule, the exposure-weighted averaged energy of Ir-192 is deduced to be around 397 keV, which falls approximately halfway between the 662 keV of Cs-137 and the average energy (146 keV) of a 250-kVp X-ray beam [9]. The above two energies are available for Farmer chamber calibrations at the NIST and ADCL in the US, and the PSDLs of other countries.

A simple averaging of the air kerma calibration factors (N_k_) of Ir-192, the recommended quantity for specifying a brachytherapy source [2, 27], can be obtained from the interpolation at the above two energies. This is a rational basis for deriving a calibration factor appropriate for Ir-192. If the chamber wall is thick enough to provide charged particle equilibrium (usually the chamber is capped with a Co-60 build-up cap), the equation for deriving the Ir-192 air kerma calibration factor (N_k_Ir_) as an average of those for 250-kVp X-rays and Cs-137 can be written as [9]:

4 $$ {\text{N}}_{K\_Ir} = \frac{{A_{W\_250kV} \times {\text{N}}_{K\_250kV} + A_{W\_Cs} \times {\text{N}}_{K\_Cs} }}{{2A_{W\_Ir} }} $$

where $$ {\text{N}}_{K\_Ir} $$$$ {\text{N}}_{K\_250kV} $$ and $$ {\text{N}}_{K\_Cs} $$ re the air kerma calibration factors of Ir-192, 250-kVp X-rays, and Cs-137 in Gy/C provided by the NIST or a PSDL, respectively; $$ A_{W\_Ir} $$$$ A_{W\_250kV} $$ and $$ A_{W\_Cs} $$ re the attenuation factors, the ratios of the exposure in the chamber to the exposure at the same point in free space, for Ir-192, 250-kVp X-rays, and Cs-137, respectively, and can be determined as outlined by Goetsch et al. [9]. The NIST (PSDL) traceable calibrations for both Cs-137 and 250-kVp X-rays should be conducted with this build-up cap present [12, 14]. The *A*_*W*_ of a radiation source X for Ir-192, 250-kVp X-rays, and Cs-137 in Eq. (4) can be calculated as:

5 $$ A_{W\_X} = 1 + T \times S_{X} $$

where *T* is the total wall thickness (in g/cm^2^) of the Farmer chamber, and $A_{W\_X}$ nd $S_{X}$ re the *A*_*W*_ and the attenuation slope, respectively, of the radiation source *X*. Based on Fig. 1 and the description in the last paragraph of Goetsch et al.’s paper (page 463) [9], $S_{X}$ the measured slope of *A*_*W*_/(graphite wall thickness), for Ir-192, 250-kVp X-rays, and Cs-137 is found to be −2.7097 × 10^−2^, −2.9032 × 10^−3^, and −2.7742 × 10^−2^ cm^2^/g, respectively. N_k_Ir_ can also be calculated using the Monte Carlo simulation procedure described by Mainegra-Hing et al. [28]. According to the research of Goetsch, Nair, and Kondo [9, 10, 29], the AKS (in Gy·m^2^·h^−1^) of Ir-192, *S*_*k*_, can be written as:

6 $$ S_{k} = R_{net} \times D^{2} \times N_{K\_Ir} \times G $$

where *D* is the source-chamber distance in meters; *G* is the non-uniformity correction factor for the Kondo-Randolph non-uniformity correction, which is equal to the “Ks^−1^” used in Kondo’s paper [29]; and *R*_*net*_ is the electrometer net reading in C/h, which is given by:

7 $$ R_{net} = R \cdot k_{ion} \cdot C_{el} \cdot C_{TP} - R_{s} $$

where *R* is the electrometer reading in C/h (better corrected with the attenuation of the applicator [13, 16]); *k*_*ion*_ is the ion recombination correction factor; *C*_*el*_ is the electrometer correction factor; *C*_*TP*_ is the temperature and pressure correction factor; and *R*_*s*_ is the room scatter in C/h, which can be acquired with 7 source-chamber distances (i.e., the 7-distance method) by curve fitting the equation:

8 $$ K = (R \cdot k_{ion} \cdot C_{el} \cdot C_{TP} - R_{s} ) \times D^{2} $$

where *K* should be a constant, since the inverse square law needs to be satisfied and all measurements were traced (by a decay constant) to the same time. *K* and *R*_*s*_ can be fitted and calculated by least squares fitting using Matlab or other fitting tools. The jig should be located at least 1 m away from any wall to ensure that *R*_*s*_ is a constant [12, 19, 30].

Following the inverse square law, non-uniformity correction is necessary because the chamber has a shape, rather than being a point. The non-uniformity correction factor *G* is equal to K·s^−1^ and can be calculated from the lookup table (Table 1 in Kondo’s paper [29]) for the regular setup, with the chamber parallel to the source. Prior to that, the user needs to calculate a shape factor, *a*/*L*, and a distance factor, *a*/*D*, where *a* is the chamber active volume radius, *L* is the half-length of the chamber active volume, and *D* is the chamber-source distance (defined above) [29]. Using a 0.6-cc Farmer chamber, an MDS calibration track stand, and a Gammamed 12i HDR unit, the experimental *G* factor (denoted *G’*) and the theoretical *G* factor (denoted *G*) were compared by Chang et al. [31]. Their results are given in the appendix (Table 3 in A1).

**Table 1:** Comparison of Ir-192 RAKR calibration coefficient traceable to ARPANSA and that calibrated at the NPL reported by Butler et al. [1]

Quantity	Symbol	Value	Uncertainty % (*k* = 2)
Interpolated air kerma calibration factor from ARPANSA	N_K_Ir_	4.890 × 10^7^ (Gy/C)	2.0
RAKR traceable to ARPANSA	N_K_ARP_	4.983 × 10^7^ (Gy/C)	2.4
RAKR measured by NPL	N_K_NPL_	4.975 × 10^7^ (Gy/C)	1.10

According to the study of Stump et al., the average differences in percentage between the AKS measured using the 7-distance technique and that using a well-type ionization chamber for the Varian VariSource (VS2000) HDR source and the new Nucletron HDR source are −0.53 ± 0.19 and −0.09 ± 0.30, respectively [12]. The combined total measurement uncertainty and the total Farmer chamber calibration uncertainty for the 7-distance technique was 2.15 % (*k* = 2) [12], which is in good agreement with the previous measurement of 2.0 % by DeWerd et al. [32].

## Modified Calibration Methods in Australia

Butler et al. conducted a comparison between the Ir-192 air kerma calibration coefficients derived at the ARPANSA using the interpolation method and that derived from the calibration at the NPL [18]. They sent a PTW 30010 Farmer chamber with a Nucletron jig to the NPL for direct calibration with the Nucletron microSelectron HDR Classic Ir-192 source (096.001) to obtain the *N*_*K_NPL*_ in Eq. (1) [18].

The air kerma calibration factor, *N*_*K_Ir*_, was calculated using Eq. (4) according to the methods of Goetsch et al. [9, 28], but modified with Co-60 instead of Cs-137 [33, 34]. Trying to follow the calibration at the NPL, Butler et al. deduced their RAKR (*N*_*K_ARP*_) by multiplying the *N*_*K_Ir*_ with a correction factor that included the corrections of the inverse square law, non-uniformity, air attenuation, scatter from room/air/jig, and catheter attenuation, following the recommendations of IAEA TecDoc 1274 [35]. The calibration results are listed in Table 1 [18].

The scale differences between *N*_*K_Ir*_ and *N*_*K_ARP*_ are mainly due to the inverse square correction. From Table 1, the differences between *N*_*K_ARP*_ and *N*_*K_NPL*_ are 0.16 %, which is trivial; however, the main difference is in calibration uncertainty, where that from ARPANSA is twice that from the NPL.

## Modified Calibration Methods in India

To calibrate the Ir-192 source, two experimental studies that used the DGMP recommendation [36], a recommendation used in German, were reported by Patel et al. and Bondel et al. [16, 17], respectively. Their Ir-192 calibrations are mainly traced from the Co-60 calibration factor of the absorbed dose to water. The calibration method is similar to the 7-distance technique (Eq. 6), but the Ir-192 air kerma calibration factor was derived differently:

9 $$ N_{K\_Ir\_DG} = (\frac{1}{1 - g}) \times (\frac{{\mu_{en} }}{\rho })_{w}^{a} \times N_{Co\_DW} \times K_{Q} $$

where *N*_*k_Ir_DG*_ is the air kerma calibration factor from the recommendation of DGMP; *g* is the fraction of energy of the secondary electrons lost in bremsstrahlung; $(\frac{{\mu_{en} }}{\rho })_{w}^{a}$ s 0.899, the mass energy absorption coefficient of air to that of water for Ir-192; *N*_*Co_DW*_ is the calibration factor of the Co-60 absorbed dose to water of the Farmer chamber; *K*_*Q*_ is the beam quality correction factor, which accounts for the differences in the energy spectrum of Co-60 for which the chamber has been calibrated and can be determined by interpolation from the energy response curves provided by the supplier or taken as 1.0, since the energy dependence of modern thimble chambers is trivial [17] (e.g., the difference between the calibration factors at the Cs-137 and 250-kVp X-ray points was approximately 2 % [12]).

The calibration results are shown in Table 2. The relative differences in percentage between the calibrated AKS and that from the manufacturer are represented as *AD*_*C*_ and *AD*_*W*_ for the calibration with a 0.6-cc Farmer chamber and a well chamber, respectively. *AD*_*C*_ and *AD*_*W*_, from the studies of Patel et al. and Bondel et al., were less than 2.1 and 1.0 %, respectively. The differences between *AD*_*C*_ and *AD*_*W*_ are less than 1.5 % for the two studies. Their uncertainties in the calibration factor obtained using the Farmer chambers were within 2.1 % (*k* = 2) and those obtained using the well chambers were slightly higher, at 2.5–3 % (*k* = 2).

**Table 2:** Calibrated AKS compared with manufacturer-specified value from Patel et al. [15] and Bondel et al. [16] obtained using 0.6-cc Farmer chamber and well chamber, with uncertainty evaluation (*k* = 2)

Calibration results	Patel et al.	Bondel et al.
AKS differences (%) for 0.6-cc chamber, AD_C_	−1.48 ± 0.50	−0.94 amber
AKS differences (%) for well chamber, AD_W_	−2.04 ± 0.37	0.21 ± 0.20
Differences between AD_C_ and AD_W_	0.56	−1.15
Uncertainty of calibration factor for 0.6-cc chamber	2.06 %	1.5 %
Uncertainty of calibration factor for well chamber	2.66 %	3.0 %

## In-air Calibration with Empirical Method

### Ir-192 Calibration Using Calibration jig

The 7-distance technique can be carried out by using only one distance setup in a room, with length *x*, width *y*, and height *z*, if the longest wall is not greater than twice the length of the shortest wall. The room will have almost the same wall surface area as that of a cubic room with size *h*, where *h* = (*xyz*)^1/3^ [14, 25]. The room scatter factor can be deduced using the empirical formula presented by Chang et al. [14]:

10 $$ R_{s} = R \cdot k_{ion} \cdot C_{el} \cdot C_{TP} \times (1 - S_{c} ) $$

where

11 $$ S_{c} \cong - \left( {a \cdot e^{ - b \cdot h} + c \cdot e^{ - d \cdot h} } \right) \times D + f \cdot e^{ - m \cdot h} + n $$

where *S*_*c*_ is the room scatter correction factor; *a* = 1.946 m^−1^, *b* = 1.472 m^−1^, *c* = 0.06998 m^−1^, *d* = 0.02036 m^−1^, *f* = 0.278, *m* = 1.56 m^−1^, *n* = 1.005; *D* (in meters) is the source-to-chamber distance (defined above); and *h* = (*xyz*) ^1/3^ in a room with length *x*, width *y*, and height *z*. Compared to the Monte Carlo calculation reported by Selvam et al. [19], this formula is accurate to within 0.3 % [14]. A user can calculate their AKS using Eq. (6) with only one source-to-chamber distance.

### Ir-192 Calibration Without Calibration jig

**Fig. 2:**
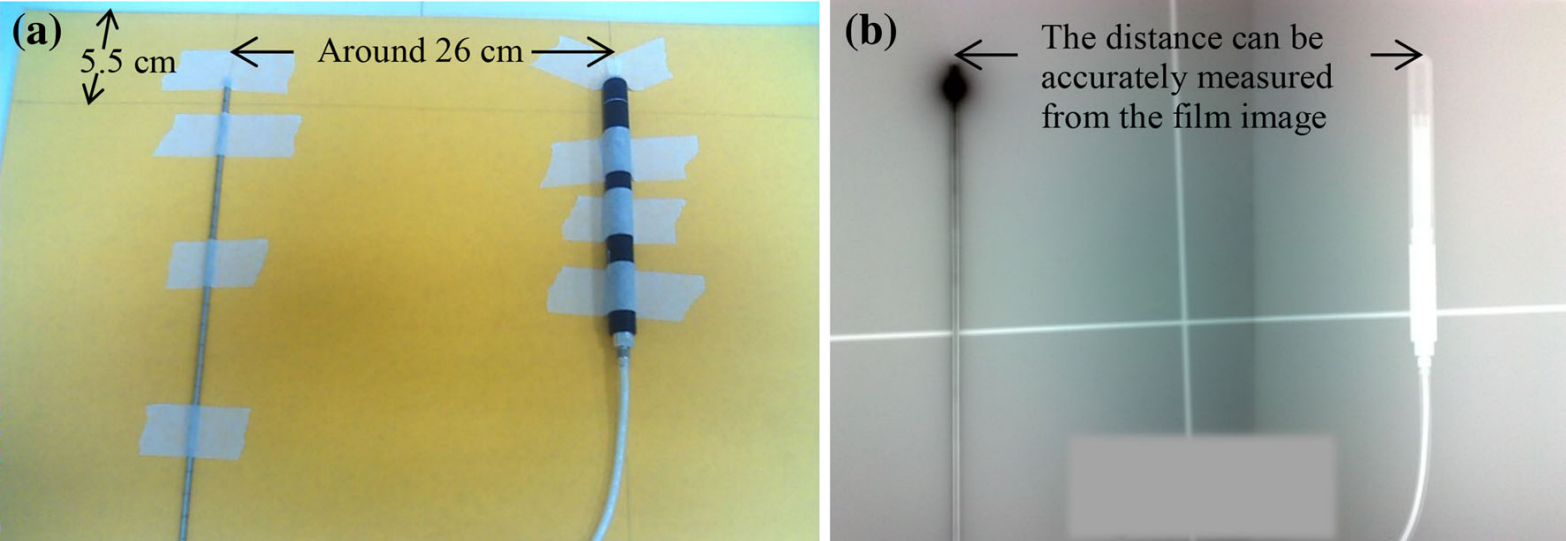
**a** Location of Farmer chamber and probe on envelope of piece of V film. **b** Developed film image

For some facilities with budgetary considerations, a physicist can accurately calibrate the Ir-192 HDR sources without using a calibration jig, but with a 0.6-cc Farmer-type ion chamber, Kodak X-Omat radiographic V film, and polystyrene solid phantoms, which are commonly employed tools for the QA of therapeutic linear accelerators [25]. To perform this calibration, one should tape a V film (25.4 × 30.5 cm^2^) on a 30 × 30 × 0.2 cm^3^ polystyrene plate. A straight applicator probe of an HDR brachytherapy unit and the Farmer-type ion chamber are affixed to the film envelope, where the probe and the chamber are parallel to each other and separated by a distance of around 26 cm (Fig. 2a). Similar to the QA of seed positioning [37], the film is then irradiated by the Ir-192 source, followed by an exposure to the simulator X-ray beam (Fig. 2b). Then, film set with the film removed is placed on the top of a 30 × 30 × 5 cm^3^ polystyrene phantom for calibration measurements. The calibration follows Eq. (6), except only one source-chamber distance is needed and Eq. (6) is rewritten as:

12 $$ S_{k} = R \cdot k_{ion} \cdot C_{el} \cdot C_{TP} \times S \times D^{2} \times N_{K\_Ir} \times G $$

where *k*_*ion*_, *C*_*el*_, *C*_*TP*-_, *N*_*K_Ir*_, and *G* are those defined in Eqs. (6) and (7); *R* is the dosimeter reading, but taken as the difference of the averaged 2-min readings and the averaged 1-min readings for several measurements; *D* is the source-chamber distance in meters calculated from the developed image on the film, which shows the structure of the chamber and a black spot from the irradiation of Ir-192; and *S* is the correction factor for the room scatter and phantom scatter, and can be written with the empirical formula [25]:

13 $$ {\text{S}} \cong (0.0478{\text{D}}^{ - 1} + 0.5S_{c}^{ - 1} + 0.5)^{ - 1} $$

Using three different 0.6-cc Farmer chambers, the calibration results reported by Chang et al. were compared to the data provided by the manufacturer and those for five different well-type ion chambers; all the differences were within 1.6 %.

## Conclusion

This paper reviewed the techniques used for the in-air calibration of Ir-192 HDR with a Farmer chamber. Most of the techniques are based on the 7-distance technique. The choices would also depend on the calibration services that the users can reach. All the reports described in this paper demonstrated that there are only slight differences between the calibration results and uncertainties obtained using well chambers and Farmer chambers. Users can get accurate calibration using the calibration services at the NPL, or more conveniently, users can calibrate the Ir-192 source with the empirical method with only one source-chamber distance.

## Appendix

See Table 3.

**Table 3:** Measured and theoretical *G* factor and their percentage differences with various source-chamber distances (*D*) reported by Chang et al. [31]

*D* (cm)	*G′* (experimental *G*)	*G* (theoretical *G*)	%Difference
46	0.998 ± 0.005	1.000	−0.22
42	1.004 ± 0.004	1.000	0.32
38	1.001 ± 0.004	1.000	0.06
34	0.997 ± 0.005	1.000	−0.34
30	0.996 ± 0.003	1.001	−0.50
28	0.998 ± 0.004	1.001	−0.25
26	0.999 ± 0.002	1.001	−0.22
24	0.999 ± 0.002	1.001	−0.18
22	1.000 ± 0.002	1.002	−0.19
20	1.002 ± 0.002	1.002	0.02
18	1.002 ± 0.002	1.002	−0.04
16	1.003 ± 0.003	1.003	0.04
14	1.008 ± 0.002	1.004	0.41
12	1.006 ± 0.001	1.005	0.08
10	1.008 ± 0.001	1.006	0.16
